# Tumor Microenvironment Activable Self‐Assembled DNA Hybrids for pH and Redox Dual‐Responsive Chemotherapy/PDT Treatment of Hepatocellular Carcinoma

**DOI:** 10.1002/advs.201600460

**Published:** 2017-01-24

**Authors:** Da Zhang, Aixian Zheng, Juan Li, Ming Wu, Zhixiong Cai, Lingjie Wu, Zuwu Wei, Huanghao Yang, Xiaolong Liu, Jingfeng Liu

**Affiliations:** ^1^ The Liver Center of Fujian ProvinceFujian Medical UniversityFuzhou350025P. R. China; ^2^ The United Innovation of Mengchao Hepatobiliary Technology Key Laboratory of Fujian ProvinceMengchao Hepatobiliary Hospital of Fujian Medical UniversityFuzhou350025P. R. China; ^3^ The Key Lab of Analysis and Detection Technology for Food Safety of the MOEFujian Provincial Key Laboratory of Analysis and Detection Technology for Food SafetyCollege of ChemistryFuzhou UniversityFuzhou350002P. R. China; ^4^ Liver Disease CenterThe First Affiliated Hospital of Fujian Medical UniversityFuzhou350005P. R. China

**Keywords:** aptamer, chemotherapy, hepatocellular carcinoma, photodynamic therapy, stimuli‐responsive

## Abstract

**Smart self‐assembled “Turn‐ON” DNA hybrids** are employed, which could respond to tumor microenvironment stimuli for cancer cell specific real‐time fluorescence imaging, tumor‐specific synergistic photodynamic therapy and chemotherapy in hepatocellular carcinoma.

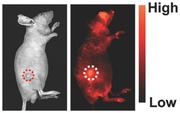

Photodynamic therapy (PDT), as a noninvasive therapeutic strategy, has been approved by the U.S. Food and Drug Administration for clinical usage to treat various tumoral diseases.^1^, ^2^, ^3^ Once exposed to appropriate light, photosensitizer (PS) as nontolerant therapeutic agent could transform endogenous oxygen to generate reactive oxygen species (ROS) then induce apoptosis of cancer cells to perform PDT.^1^ Chlorin e6 (Ce6), as an efficient photosensitizing (PS) agent, was widely applied for PDT because of rapid elimination from the body, high singlet oxygen generation efficiency and activation by NIR light.^4^, ^5^, ^6^, ^7^ However, the nonspecific damage to vascular system and normal tissues once the injected PS exposed to sun light, can make serious side effects in patients including burn, swelling, and pain. In addition, the ROS generation of PS would rapidly consume tissue endogenous oxygen to produce severe hypoxia, while has no succeeding oxygen supply due to incomplete nature of the tumor vasculature, which could significantly hinder the PDT efficacy. Recently, tumor microenvironment triggered drug delivery system (DDS) has captured extensive attention due to their smart and finely controllable response, which could reduce the drug side effects and further increase their anticancer efficiency.^8^, ^9^, ^10^ Thus, design a stimuli‐responsive DDS that combined PDT with controlled drug release behavior could be an effective strategy to avoid side‐effects of PDT and increase the antitumor efficiencies.

Nevertheless, the precise targeting of tumor cells with minimizing side effects in normal cells is a prerequisite to DDS for cancer treatment. Structured single‐stranded DNA such as aptamer with low molecular weight is a promising molecular targeting element that has been extensively designed for targeted drug delivery applications, due to its advantages in terms of small size, synthetic accessibility, thermally stable, low immunogenicity, and flexible chemical modification.^11^, ^12^, ^13^ Additionally, aptamer was not only could act as targeting ligand but also could be applied as drugs delivery system for diagnosis and targeted therapy.^14^, ^15^ Herein, we designed a smart Ce6‐DOX‐DNA hybrid (named as Ce6‐fDNA^Dox^ probe) with tumor microenvironment responsive “Turn‐ON” properties to perform cancer cell specific real‐time fluorescence imaging, as well as increase the specificity of photodynamic therapy and chemotherapy. The hybrids were self‐assembled by three types of “components” via complementary base pairing, and subsequently loaded with DOX (Doxorubicin) for real‐time NIR fluorescence imaging, tumor‐specific photodynamic therapy and chemotherapy via redox‐responsive ROS generation, and pH‐triggered DOX release in HCC (Hepatocellular Carcinoma). As illustrated in **Scheme** **1**, the self‐assembled Ce6‐fDNA^DOX^ probe was prepared by hybridization using three types of functionalized single‐strained DNA (f‐ssDNA), termed as redox‐responsive quencher ssDNA (RQD), Ce6 labeled ssDNA (CD), and cell‐targeting aptamer “TLS11a” (TD), respectively, and each strand has a “sticky end” segment to assemble each other through a complementary base pairing with uniform size. The sequence information of these ssDNA was shown in **Table** **1**. For selectively recognizing the hepatocellular carcinoma cells, the aptamer “TLS11a,” which could specifically recognize the membrane surface of HCC cells,^16^, ^17^, ^18^, ^19^ was incorporated into “TD” chain. To achieve tunable switch “ON” for real‐time fluorescence imaging and PDT treatment specifically in cancer cells with minimal side effects, we incorporated the BHQ2 quencher group to the 3′ end of RQD chain with a disulfide linkage; after the self‐assembling of these single‐strained DNAs, the fluorescence and ROS generation ability of Ce6 would be quenched by BHQ2 due to the energy transfer in normal cells; the BHQ2 quencher could be released due to the cleavage of disulfide linkage by the high level of GSH in cancer cells, then the fluorescence and the ROS generation ability of Ce6 could be recovered for NIR imaging and PDT treatment of cancer cells. Meanwhile, the loaded DOX in Ce6‐fDNA probe through π–π stacking could be released due to the slightly acidic conditions of cancer cells and its lysosomes to perform chemotherapy, which then could combined with the “on” state of PDT for synergistic treatment of HCC.

**Scheme 1:**
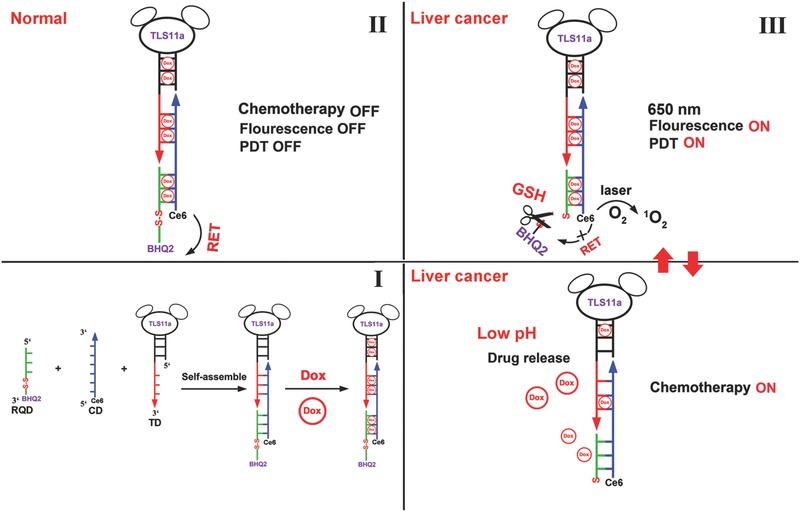
Schematic view of the preparation (I) and functional principle (II, III) of Ce6‐fDNA^Dox^ probe. The self‐assemble procedure of Ce6‐fDNA^Dox^ probe was shown in step (I). In normal cells, the BHQ2 quencher at one end of Ce6‐fDNA^Dox^ probe would quench the fluorescence and ROS generation ability of Ce6, and the probe would be in “OFF” state due to normal level of redox (II). Responding to the high redox condition of cancer microenvironment, the BHQ2 quencher would release from the probe end, which could recover the fluorescence and ROS generation ability of Ce6 for PDT therapy, and the DOX could release from the Ce6‐fDNA^Dox^ probe for chemotherapy due to the low pH condition in cancer cells and lysosomes (III).

**Table 1:** Sequences of the synthesized functional ssDNA strand (5′‐3′)

Name	Sequences
RQD	GTCCTCTCACGTGCGGTCTGTCGAGGCCTGC(S‐S)GGC‐BHQ2
CD	NH_2_‐GCAGGCCTCGACAGACCGCACGTGAGAGGACGAGCTCGGCGAGCGCTGCCAGTGA
TD	CAGCATCCCCATGTGAACAATCGCATTGTGATTGTTACGGTTTCCGCCTCATGGACGTGC
	TGTCACTGGCAGCGCTCGCCGAGCT
NTD	AAGTGCATGAGTGAGAGGACTACGTCTGCTGACTGCGAGCCAGTGAGATGAGCGAGGCG
	ATTCACTGGCAGCGCTCGCCGAGCT

As a proof of concept, we chose 24‐bases sticky ends to obtain a stable enough self‐assembled structure and load the DOX in this paper. To demonstrate the precise self‐assemble of these three functionalized ssDNA, agarose gel electrophoresis was used to examine the formation of each building unit. As shown in Figure S1 (Supporting Information) and **Figure** **1**A, we could clearly see that the three types of functionalized ssDNA were indeed completely self‐assembled into new high‐molecular weight products (Ce6‐fDNA) in Lane 7 with the molar ratio of RQD, CD, and TD as 1:1:1, according to the gradually increasing of the product's molecular weight. Meanwhile, the Ce6 labeling of CD chain did not affect the complementary base pairing and the self‐assemble efficiency of Ce6‐fDNA probe (as shown in Figure 1A).

**Figure 1:**
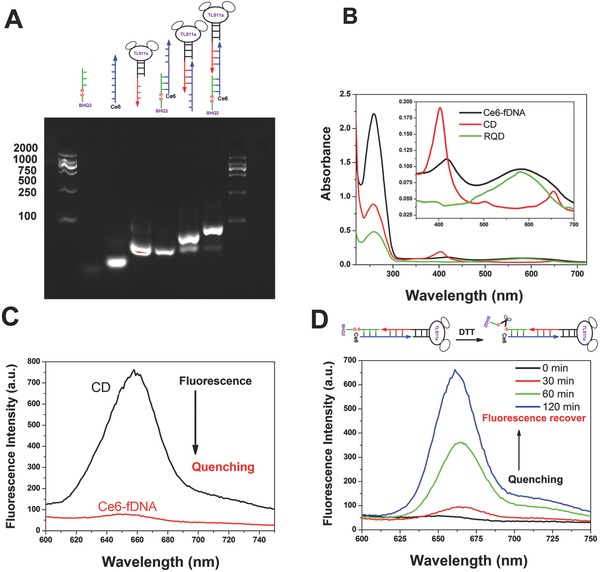
(A) Lanes 1 and 8 are the DNA marker, lanes 2–4 are RQD, CD, and TD, respectively. Lanes 5 and 6 are RQD + CD and CD + TD, respectively. Lane 7 is the self‐assembled final products containing RQD, CD, and TD. B) Vis–NIR spectra of RQD, CD, and Ce6‐fDNA probe. C) Fluorescence spectra of CD chain and Ce6‐fDNA probe. D) Fluorescence emission spectrum of Ce6‐fDNA probe (excitation at 405 nm) in the presence of DTT (10 × 10^−3^ m).

To confirm the successful assembling of Ce6‐fDNA probe, UV–vis spectra of CD chain, RQD chain, and Ce6‐fDNA probe was analyzed. As shown in Figure 1B, comparing with the CD chain (red line) and RQD chain (green line), the Ce6‐fDNA probe (black line) exhibited two absorption peaks at 420 nm corresponding to the characteristic absorption peak of Ce6 at 405 nm with slightly red‐shift that was consistent with previous reported works,^12^ and at 585 nm corresponding to the characteristic absorption peak of BHQ2. In addition, the fluorescence intensity of Ce6 in Ce6‐fDNA probe was significantly quenched roughly 9.4‐folds (89.4%) by the nearby BHQ2, which was calculated from the emission peak at 675 nm, compared with the same amount of CD chain (Ce6 concentration) in TM buffer, due to the efficient energy transfer between Ce6 and BHQ2 (Figure 1C and Figure S2, Supporting Information). To further confirm whether reducing agent could specifically cleave the disulfide linker at the 5′ end of Ce6‐fDNA probe, which could recover the florescence of Ce6, 5 × 10^−6^ m of Ce6‐fDNA probe was incubated with or without 10 × 10^−3^ m dithiothreitol (DTT) in TM buffers at room temperature, respectively. As shown in Figure 1D and Figure S3 (Supporting Information), the fluorescence intensity of Ce6‐fDNA probe was gradually increased along with time after adding DTT to cleave the disulfide linker, and the fluorescence intensity was increased more than tenfolds after 2 h comparing with the beginning (Figure 1D and Figure S4, Supporting Information). In contrast, no obvious fluorescence increase was detected in Ce6‐fDNA probe without DTT incubation after 2 h. Furthermore, the singlet oxygen generation ability of Ce6‐fDNA probe was investigated through using the ROS indicator (anthracenediylbis (methylene) dimalonic acid, ABDA).^4^ Due to the efficient quenching of Ce6 by nearby BHQ2 inside the Ce6‐fDNA probe, there is no significant decrease of the ABDA absorbance upon laser irradiation (670 nm, 0.2 W cm^−2^, as shown in Figure S5, Supporting Information), which clearly indicates the suppression of singlet oxygen production by energy transfer between Ce6 and BHQ2. However, the ABDA absorbance showed a sharp decline along with time when Ce6‐fDNA probe incubated with DTT and then irradiated with 670 nm laser (0.2 W cm^−2^, **Figure** **2**A,B), due to the cleavage of disulfide linker and release the quencher BHQ2, which could recover the singlet oxygen production of Ce6. Afterward, the chemotherapy drug “DOX” was loaded into the Ce6‐fDNA probe in TM buffer through π–π interaction within “GC” and “CG” double helix to obtain the final products “Ce6‐fDNA^DOX^ probe.” To confirm DOX payload, both UV–vis spectra and fluorescence spectra were analyzed. As shown in Figure 2C,D, the Ce6‐fDNA^DOX^ probe (black line) exhibited a new absorption peak at 505 nm, in accordance with the characteristics absorption peak of DOX; meanwhile, the fluorescence of DOX (black line) was significantly quenched in Ce6‐fDNA^DOX^ probe, compared with the free DOX (red line), with a quenching efficiency of 89.41% (Figure S6, Supporting Information) due to the aggregation of DOX inside the probe. Furthermore, the loaded DOX could be fast released from the Ce6‐fDNA^DOX^ probe by responsive to the external low pH. As shown in Figure S7 (Supporting Information), DOX fluorescence of Ce6‐fDNA^DOX^ probe was significantly increased at pH 5.0, compared with Ce6‐fDNA^DOX^ probe at pH 7.0, which was attributed to the increased hydrophilicity and solubility of DOX at this pH.^20^

**Figure 2:**
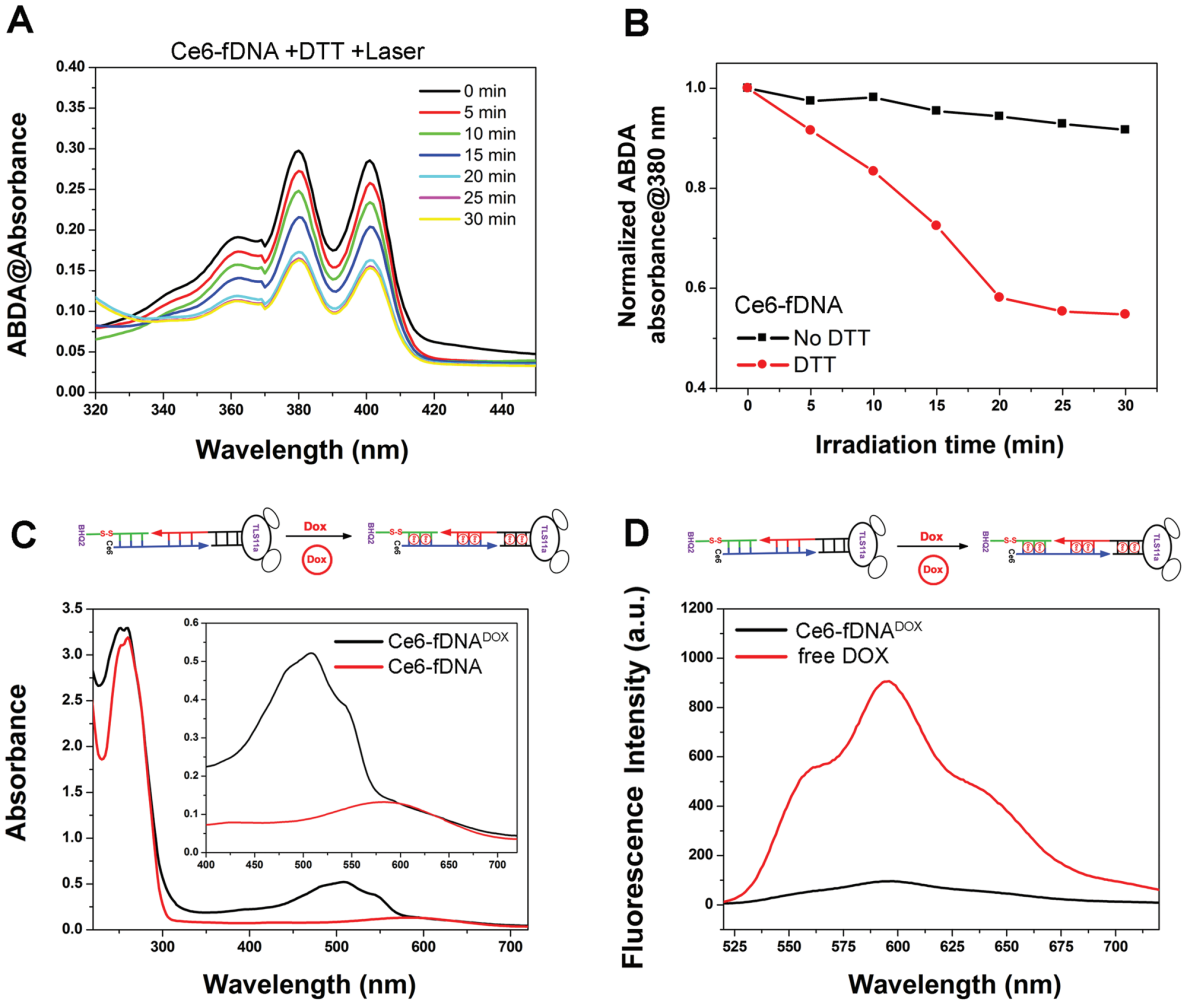
A) The absorbance of 9,10‐dimethylanthracene (ABDA, 100 × 10^−6^ m) after photodecomposition by ROS generation upon 670 nm laser irradiation at 0.2 W cm^−2^ in the presence of Ce6‐fDNA probe with DTT (10 × 10^−3^ m) in PBS solution. B) Normalized absorbance of the 9,10‐imethylanthracene (ABDA, 100 × 10^−6^ m) at 380 nm during photodecomposition by ROS generation of Ce6‐fDNA probe in TM buffer upon 670 nm laser irradiation (0.2 W cm^−2^) without DTT (black line) or in the presence of DTT (10 × 10^−3^ m), respectively. C) Vis–NIR spectra of Ce6‐fDNA^DOX^ probe and Ce6‐fDNA probe in TM buffer. D) Fluorescence spectra of free DOX and Ce6‐fDNA^DOX^ probe in TM buffer.

Active targeting and selectively internalized into cancer cells are of great importance for minimizing toxicity to normal cells. The HCC cell specific targeting ability of our Ce6‐fDNA probe and Ce6‐fDNA^DOX^ probe were further examined. As shown in **Figure** **3**A, the Ce6‐fDNA probe could be selectively internalized into HepG2 cells, but not Hela cells (only with minimal background nonspecific uptake) observed by confocal laser scanning microscopy analysis (CLSM), due to the specific HCC cell targeting ability of aptamer “TLS11a” in the TD chain. Meanwhile, the Ce6 fluorescence signals of Ce6‐fDNA probe could gradually increase along with time inside the cytoplasm of HepG2 Cells, because of the cleavage of disulfide linker by intracellular GSH. Furthermore, the controlled release of DOX from Ce6‐fDNA^DOX^ probe was further analyzed by CLSM. The HepG2 cells were first incubated with 5 × 10^−6^ m Ce6‐fDNA^DOX^ probe for 2 h, then the free probes were removed and the cells were further incubated with fresh culture medium for additional 2 h. As shown in Figure 3B, the fluorescence of DOX (ex 488 nm, em 595 nm) and Ce6 (ex 633 nm, em 650 nm) were clearly detected after the 2 + 2 h incubation in HepG2 cells, respectively; although the DOX and Ce6 fluorescence also could be detected in Hela cells under the same conditions, these fluorescence signals are much lower than that in HepG2 cells, which is due to the specific internalization of the Ce6‐fDNA^DOX^ probe in HepG2 cells but only nonspecific background uptake in Hela cells. In HepG2 cells, the Ce6 fluorescence (red) is mainly diffused in cytoplasm due to the fluorescence recover, while partial of the DOX fluorescence (green) is appeared in the nucleus of HepG2 cells due to the DOX release from Ce6‐fDNA^DOX^ probe and subsequently enter into the cell nucleus. To further confirm the controlled release behavior of Ce6‐fDNA^DOX^ probe, HepG2 cells were incubated with Ce6‐fDNA^DOX^ probe or Ce6‐fNDNA^DOX^ (the probe without the specific HCC cell targeting element) for 2 h, then washed and incubated with fresh culture medium for additional 2, 4, 6, and 24 h, respectively. The amount of released DOX was evaluated by recording the fluorescence intensity of the whole cells in 96 wells (*n* = 5). As shown in Figure S8 (Supporting Information), the fluorescence intensity of DOX released from Ce6‐fDNA^DOX^ probe (ex 488, em 595 nm) was presented a concomitant increasing manner through expanding the incubation time. Furthermore, the fluorescence intensity of DOX from Ce6‐fDNA^DOX^ was significantly higher than that of Ce6‐fNDNA^DOX^, indicating that the Ce6‐fDNA^DOX^ probe could be more efficiently uptaken by HepG2 cells due to the specific recognizing, and could serve as an effective drug delivery system for selective targeting HepG2 cells with controlled drug release properties.

**Figure 3:**
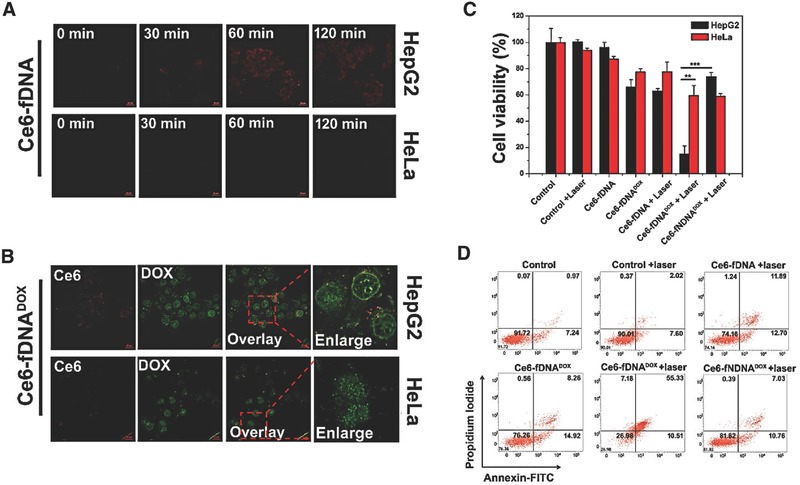
A) HepG2 cells or HeLa cells incubated with Ce6‐fDNA (Ce6, 5 × 10^−6^ m) probe for 0, 30, 60, and 120 min, respectively (scale bar: 20 µm). B) HepG2 cells or HeLa cells incubated with Ce6‐fDNA^DOX^ (Ce6, 5 × 10^−6^ m; DOX, 96.3 × 10^−6^ m) probe for 2 + 2 h (scale bar: 20 µm). C) Cell viability of HepG2 cells and HeLa cells treated with Ce6‐fDNA, Ce6‐fDNA^Dox^, and Ce6‐fNDNA^Dox^ with or without 670 nm laser irradiation (*n* = 5). The statistical analysis was performed with the two‐tailed paired Student's *t*‐test, **p < 0.01, ***p < 0.001 (Ce6, 2.05 × 10^−6^ m; DOX, 39.5 × 10^−6^ m). D) Apoptosis of HepG2 cells incubated with Ce6‐fDNA, Ce6‐fDNA^Dox^, and Ce6‐fNDNA^Dox^ with or without PDT treatment (670 nm, 0.2 W cm^−2^), respectively. The cells apoptosis were determined by flow cytometry analysis using Annexin V‐FITC and PI staining (Ce6, 2.05 × 10^−6^ m; DOX, 39.5 × 10^−6^ m).

Since the Ce6‐fDNA^DOX^ probe has both the redox responsive PDT effects and pH responsive chemotherapy effects, the synergistic antitumor efficacy of Ce6‐fDNA^DOX^ was further investigated by using a Cell Counting Kit‐8 (CCK8) assay. As shown in Figure 3C, under the 670 nm laser irradiation with the power intensity of 0.2 W cm^−2^, Ce6‐fDNA (Ce6, 2.05 × 10^−6^ m) inhibited 37.9% of the proliferation; Ce6‐fDNA^DOX^ (Ce6, 2.05 × 10^−6^ m; Dox, 39.5 × 10^−6^ m) without laser irradiation inhibited 33.9% of the proliferation; however, Ce6‐fDNA^DOX^ with 670 nm laser irradiation significantly inhibited 85.1% of cell proliferation. In contrast, the equivalent Ce6‐fNDNA^DOX^ (Ce6, 2.05 × 10^−6^ m; Dox, 39.5 × 10^−6^ m) under the same condition only inhibited 26.1% of cell growth, which due to none sufficient probe uptake. These results clearly demonstrated remarkable increased synergistic antitumor effects of our Ce6‐fDNA^DOX^ through PDT and chemotherapy in HepG2 cells. Furthermore, the synergistic antitumor efficacy of Ce6‐fDNA^DOX^ probe was determined by flow cytometry using Annexin‐V‐fluoroisothiocyanate (FITC)/propidium iodide(PI) staining. The stained cells were divided into four subgroups, the viable group, the early apoptotic group, the late apoptotic/necrotic group, and the dead cells/debris group, which were localized in the lower left, lower right, upper right, and upper left quadrants, respectively. As shown in Figure 3D, the majority of HepG2 cells were localized in the lower left quadrant with more than 91.72% of the viable cells in the control, and the percentage of apoptotic and necrotic cells was only 8.28% for the control group. Meanwhile, compared with the control, most of the cells without Ce6‐fDNA^DOX^ treatment still remained alive (90.01% of the viable cells) under the laser irradiation at 670 nm (0.2 W cm^−2^). However, the percentage of viable cells was significantly decreased (26.98% of the viable cells), and the percentage of apoptotic and necrotic cells was obviously increased to 73.02% in the Ce6‐fDNA^DOX^ treated groups with laser irradiation, which was much higher than PDT treatment alone (25.84%, only incubated with Ce6‐fDNA with laser irradiation) or chemotherapy alone (23.74%, only incubated with Ce6‐fDNA^DOX^ without laser irradiation). In contrast, the percentage of apoptotic and necrotic cells was only 18.18% for the Ce6‐fNDNA^DOX^ group under laser irradiation, which may be due to the insufficient amount of Ce6‐fNDNA^DOX^ by nonspecific uptake. These results suggested the selectivity and combined cell killing efficiency or the combined PDT and chemotherapy effects of our Ce6‐fDNA^DOX^.

To further investigate the Ce6‐fDNA^DOX^ targeting ability in *vivo*, a systematic comparative investigation was performed to validate the selective accumulation of Ce6‐fDNA^DOX^ in HepG2‐tumor bearing mice model. As shown in **Figure** **4**A, a weak red fluorescence signal of Ce6 from Ce6‐fDNA^DOX^ could be seen in the tumor site (white ring) in the first 1 h after intravenous injecting of Ce6‐fDNA^DOX^ into mice, followed by a significant increase of the Ce6 fluorescence signal at the tumor site post 2 h of injection. However, the Ce6 fluorescence signal was obviously decreased after 4 h of injection. In contrast, no Ce6 fluorescence signal from Ce6‐fNDNA^DOX^ could be seen at the tumor site during the entire procedure after the injection of Ce6‐fNDNA^DOX^ into HepG2‐tumor bearing mice model (Figure 4A). Moreover, the biodistribution of Ce6‐fDNA^DOX^ or Ce6‐fNDNA^DOX^ in HepG2‐tumor bearing mice model was also examined after 2 h of injection (Figure 4B). It could be clearly seen that the tumor from Ce6‐fDNA^DOX^ injected mice presented significantly higher fluorescence signal of Ce6 (red) and DOX (green) than that of Ce6‐fNDNA^DOX^ injected mice, and the Ce6‐fDNA^DOX^ injected mice presented more accumulation in tumor than heart, lung, or spleen. These results clearly suggested that our Ce6‐fDNA^DOX^ had specific tumor targeting ability for hepatocellular carcinoma in vivo.

**Figure 4:**
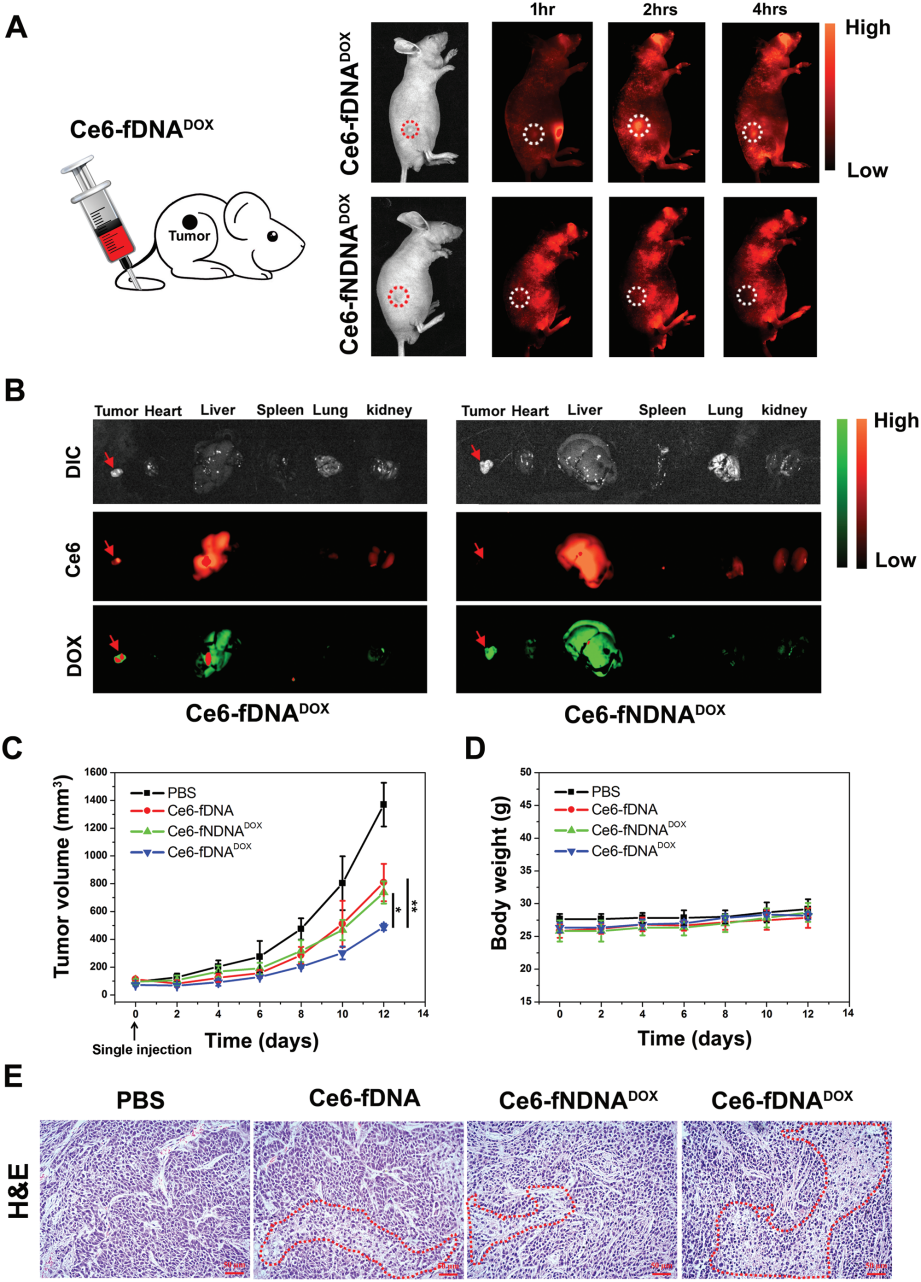
A) HepG2 tumor‐bearing mice were intravenously injected with Ce6‐fDNA^DOX^ or Ce6‐fNDNA^DOX^ (with single injection) and then imaged with ChemiDoc MP Imaging System. Timelapse fluorescence images of Ce6 in vivo after injection of Ce6‐fDNA^DOX^ (upper panel) or Ce6‐fNDNA^DOX^ (lower panel) (Ce6, 5.12 × 10^−6^ m; DOX, 98.87 × 10^−6^ m). B) Optical and fluorescence images of different organs that isolated from HepG2‐tumor bearing mice after 2 h of Ce6‐fDNA^DOX^ or Ce6‐fNDNA^DOX^ injection (Ce6: red fluorescence; DOX: green fluorescence). C) Tumor volume of mice (*n* = 5) after indicated treatments (the treatment was taken place at day 0) as follows: intravenously injected (with single injection) with PBS, Ce6‐fDNA, Ce6‐fNDNA^DOX^, and Ce6‐fDNA^DOX^ (Ce6, 5.12 × 10^−6^ m; DOX, 98.87 × 10^−6^ m), then irradiated with 670 nm laser (0.2 W cm^−2^) for 10 min after 2 h of injection, and the statistical analysis was performed with the two‐tailed paired Student's *t*‐test, *p < 0.05, **p < 0.01. D) Mean body weight of mice that received the indicated treatments (*n* = 5). E) Representative images of H&E staining from tumors after 48 h of the indicated treatments, the necrotic area is indicated by red dashed (50 µm).

Next, we evaluated the in vivo synergistic antitumor efficacy of Ce6‐fDNA^DOX^ in HepG2‐tumor bearing mice model. After dorsal tumor nodules grew to ≈100 mm^3^, mice were randomly divided into four groups for studying the therapeutic efficacy; changes in tumor volumes of these four groups that received various treatments as indicate (as mentioned in the Experimental Section) were monitored for 12 d. As shown in Figure 4C,D, mice experienced a rapid tumor growth in PBS buffer (phosphate buffered saline) treated mice with 670 nm laser irradiation for 10 min (0.2 W cm^−2^), indicating that only the use of laser irradiation has no therapeutic effects. In contrast, Ce6‐fDNA, Ce6‐fNDNA^DOX^, and Ce6‐fDNA^DOX^ administration/irradiation groups showed obvious delay of tumor growth. Moreover, the mice received Ce6‐fDNA^DOX^ treatment with 670 nm laser irradiation exhibited significant slower tumor growth, compared with the mice that received Ce6‐fDNA treatment with 670 nm laser irradiation, which due to the synergistic therapeutic effects of PDT and chemotherapy. Noteworthy, the Ce6‐fDNA^DOX^ treatment had much better therapeutic effects than the Ce6‐fNDNA^DOX^ treatment since the Ce6‐fDNA^DOX^ probe could more efficiently accumulate in the tumor site due to the targeting ability of TLS11a aptamer. Taken together, these results clearly demonstrated that our Ce6‐fDNA^DOX^ probe could selectively target to HepG2 tumor cells in vivo to perform the synergistic PDT and chemotherapy with excellent therapeutic efficacy.

High drug toxicity usually leads to weight loss, we thus further measured the body weight of HepG2‐tumor mice model for all groups during the treatment to evaluate the toxicity of our probe. As shown in Figure 4D, no obvious weight loss of all groups was observed, indicating that the toxicity or side effects of our Ce6‐fDNA^DOX^ probe was not serious at all. Moreover, we also evaluated the tumor tissues by hematoxylin and eosin (H&E) after 48 h of indicated treatment (Figure 4E). Compared with PBS treated group, the tumors that received Ce6‐fDNA treatment with 670 nm laser irradiation or Ce6‐fNDNA^DOX^ treatment with 670 nm laser irradiation showed a certain degree of tissue and cellular damage, due to the apoptosis of cancer cells induced by the PDT or nonspecific accumulation of Ce6‐fNDNA^DOX^ for limited PDT and chemotherapy. However, the Ce6‐fDNA^DOX^ treated tumors with 670 nm laser irradiation showed obvious cell destruction and damaged areas, as indicated by decrease general intensity of cancer cells. These results furhter proved the significantly improved synergistic therapeutic efficiency of PDT/chemotherapy of our Ce6‐fDNA^DOX^ probe.

In summary, we reported an activable self‐assembled DNA hybrid system (Ce6‐fDNA^DOX^ probe) for real‐time NIR fluorescence imaging, tumor‐specific photodynamic and chemotherapy in vitro and in vivo. In this system, the smart Ce6‐fDNA^DOX^ probe was able to overcome the side‐effects by switching “ON” and “OFF” of the PDT as required, and enhance the treatment outcomes by combing with synergistic chemotherapy.

## Experimental Section

*Materials*: All oligonucleotides were synthesized by Sangon Biotech Company and purified by using reversed phase HPLC. Chlorin e6 (Ce6), *N*‐(3‐dimethylamino‐propyl)‐*N*‐ ethylcarbodiimide hydrochloride, *N*‐hydroxy succinimide (NHS), 9,10‐ ABDA, and BSA (Bovine Serum Albumin) were purchased from Sigma‐Aldrich. CCK8 and Annexin V‐FITC apoptosis detection kit were purchased from Dojindo Laboratories (Kumamoto, Japan). Deionized water with a resistivity of 18.2 MΩ cm was obtained from a Milli‐Q Gradient System (Millipore, Bedford, MA, USA) and used for all experiments. Unless specified, all other chemicals were commercially available and used as received.

*Cell Culture*: HepG2 cells (hepatocellular carcinoma) and HeLa cells (human cervical epithelioid carcinoma) were purchased from ATCC (Manassas, VA). All cells were cultured in RPMI 1640 medium (ATCC, Manassas, VA) supplemented with 10% fetal bovine serum (FBS) and 100 IU mL^−1^ penicillin–streptomycin (Cellgro, Manassas, VA).

*Preparation of Ce6‐fDNA Probe*: First, an equimolar of *N*‐hydroxysu ccinimide ester (NHS), dicyclohexyl carbodiimide, and Ce6 were dissolved in anhydrous DMF (N,N‐Dimethylformamide) in the dark for 30 min. Activated Ce6 was then added to 5′ end amine modified ssDNA strands (CD) in NaHCO_3_ at pH 7 by vigorously stirring overnight in the dark. The unconjugated Ce6 was removed by ethanol precipitation of DNA, and repeated for four times. Quantification of the conjugated DNA and Ce6 was done by measuring the absorbance at 260 and 404 nm. The standard curve has a very good linear relation with Ce6 from the concentration of 1–25 µg mL^−1^ (*Y* = 0.0553× + 0.0547, *R* ^2^ = 0.9975).

Second, 3′ end disulfide and BHQ2 comodified ssDNA strands (RQD), TLS11a aptamer (TD), and none targeting aptamer (NTD) were synthesized by Sangon Biotech Company. Then, stoichiometric quantities of the three kinds of ssDNA strands: RQD chain, photosensitive chain (CD), and cell‐targeting aptamers (TLS11a, TD) or NTDs) were separately added to three Eppendorf tubes with a buffer solution containing TM buffer (20 × 10^−3^ m Tris, 10 × 10^−3^ m MgCl_2_, pH 7.5). Binding buffer was prepared with D‐PBS supplemented with 4.5g L^−1^ of glucose, 5 × 10^−3^ m of MgCl_2_, 0.1 mg mL^−1^ of yeast *t*RNA, and 1 mg mL^−1^ of BSA. The ideal molar ratio of RQD to CD to TD (or NTD) is 1:1:1. Then each mixture was heated to 95 °C for 5 min and cooled down to room temperature over 4 h to form the desired self‐assemble of Ce6‐fDNA probe or Ce6‐fNDNA probe (10 × 10^−6^ m).

*Preparation of Ce6‐fDNA^DOX^ Probe*: Certain amounts of DOX were added into the 10 × 10^−6^ m Ce6‐fDNA probe or Ce6‐fNDNA probe. Then, the mixture was coincubated with shaking for 12 h, and the free DOX was removed by ethanol precipitation of Ce6‐fDNA^DOX^ probe or Ce6‐fNDNA probe (repeated for four times). The amount of loaded DOX was determined by measuring the absorbance at 500 nm in TM buffer (Beijing Perkinje General Instrument Co., China). The standard curve has a very good linear relation with DOX from the concentration of 1–25 µg mL^−1^ (*Y* = 10.163× + 0.0174, *R* ^2^ = 0.9946).

*Agarose Gel Electrophoresis*: Each DNA sample (10 µL) was mixed with 6× loading dye (2 µL) and analyzed using 3% agarose gel at 100 V for about 30 min in 1× TBE buffer (89 × 10^−3^ m tris (hydroxymethyl) aminomethane, 2 × 10^−3^ m ethylenediaminetetraacetic acid and 89 × 10^−3^ m boric acid, pH 8.0). The bands were stained with SYBR green, visualized by UV illumination (312 nm), and photographed by “ChemiDoc MP Imaging System” from Bio‐rad.

*Redox‐Responsive Fluorescence Imaging of Ce6‐fDNA Probe*: 5 × 10^−6^ m of Ce6‐fDNA probe was incubated with 10 × 10^−3^ m DTT for different times. The fluorescence spectra were recorded at room temperature in a quartz cuvette on a FluoroMax‐4 spectrofluorometer (HORIBA, NJ, USA). The excitation wavelength was 405 nm, and the emission wavelengths were in the range from 650 to 750 nm with both excitation and emission slits of 10 nm under a PMT voltage of 950 V.

*ROS Generation of Ce6‐fDNA Probe under 670 nm Laser Irradiation*: ROS generation of Ce6‐fDNA probe was measured through using ABDA as an indicator.^4^, ^15^ Briefly, the Ce6‐fDNA probe in water containing 10 × 10^−3^ m dithiothreitol was incubated for 4 h, then the ethanol precipitated Ce6‐fDNA probe was added into the 100 × 10^−6^ m ABDA solution under the 670 nm laser irradiation with the power intensity of 0.2 W cm^−2^ for 0, 5, 10, 15, 20, 25, and 30 min, respectively; afterward, the absorbance change of ABDA was measured by an UV–vis spectrometer (Beijing Perkinje General Instrument Co., China).

*Real‐Time Fluorescence Imaging of HCC Cells In Vitro*: The selective uptake of Ce6‐fDNA and Ce6‐fDNA^Dox^ probe by HepG2 cells and HeLa cells were investigated using confocal microscopy, respectively. HepG2 cells (5 × 10^4^) or HeLa cells (3 × 10^4^) were seeded onto 35 mm glass‐bottom Petri dishes and cultured for 24 h at 37 °C in the incubator. Then the original medium was replaced with fresh culture medium containing Ce6‐fDNA probe, and further incubated for 0, 30, 60, and 120 min, respectively. Subsequently, the HepG2 cells or Hela cells were washed three times with PBS (pH 7.4) at room temperature. Finally, the cells were imaged by a confocal microscope (LSM 780, USA) with 405 nm laser excitation for Ce6. Second, Ce6‐fDNA^DOX^ was added into the cells for 2 h, and then the original medium was replaced with fresh culture medium for an additional 2 h. Finally, the cells were imaged by a confocal microscope (LSM 780, USA) with 488 nm laser excitation for DOX (em 550–590 nm) and 405 nm laser excitation for Ce6 (650–680 nm).

*Synergistic Antitumor Efficacy of Ce6‐fDNA^DOX^ Probe In Vitro*: Cell Counting Kit (CCK8) was used to study the photodynamic/chemotherapy cell toxicity of Ce6‐fDNA (Ce6, 2.05 × 10^−6^ m), Ce6‐fDNA^DOX^ (Ce6, 2.05 × 10^−6^ m; DOX, 39.5 × 10^−6^ m), and Ce6‐fNDNA^DOX^ (Ce6, 2.05 × 10^−6^ m; DOX, 39.5 × 10^−6^ m) against HepG2 cells and HeLa cells. The cells were seeded in a 96‐well plate at a density of 1 × 10^4^ cells per well and incubated in a humidity atmosphere (with 5% CO_2_) for 24 h. Then the original medium was replaced with fresh culture medium containing Ce6‐fDNA, Ce6‐fDNA^DOX^ or Ce6‐fNDNA^DOX^. Meanwhile, the cells incubated with cell culture medium only were prepared as untreated control. The medium was aspirated after 4 h incubation, then were washed twice with 200 µL PBS solution at room temperature to remove noninternalized probes. Afterward, fresh culture medium was added, and the cells were exposed to 670 nm laser irradiation (0.2 W cm^−2^) for 10 min. After laser irradiation, the cells were incubated with fresh RPMI‐1640 culture medium containing 10% FBS at 37 °C for 48 h. Cell viability was expressed as follows: Cell viability (%) = (ODsample ‐ ODblank)/(ODcontrol ‐ ODblank) × 100%. The ODsample and ODcontrol are the absorbance values of the treated cells (as indicated) and the untreated control cells (without nanoparticles), respectively. The ODblank was the absorbance of CCK8 reagent itself at 450 nm. All experiments were performed in quadruplicate.

*Flow Cytometry Evaluation of Cell Apoptosis Induced by Ce6‐fDNA^Dox^ Probe*: The Annexin‐FITC/PI staining method is used to study drug induced cell death. HepG2 cells was first seeded into a 6‐well plate at a density of 1 × 10^5^ cells per well at 37 °C in a 5% CO_2_ atmosphere for 24 h. The cells then were washed three times with PBS to remove dead cells, followed by incubation with Ce6‐fDNA^Dox^ (or the probe as indicated) that were dispersed in a culture medium at 37 °C for 4 h. Next, the cells were washed by PBS to remove the non‐uptaken Ce6‐fDNA^Dox^ probe, and then exposed to 670 nm laser irradiation (0.2 W cm^−2^) for 10 min. Afterward, the cells were incubated with fresh culture medium at 37 °C for 48 h. Then, the cells were collected and resuspended in 500 µL binding buffer, and the Annexin V‐FITC and PI were added following the manufacturer's recommendation. Afterward, samples were incubated in darkness for 15 min at room temperature and then analyzed using flow cytometry.

*Fluorescence Imaging and Synergistic Antitumor Efficacy of Ce6‐fDNA^Dox^ In Vivo*: Male BALB/c‐nude mice (six weeks old) were purchased from China Wushi, Inc. (Shanghai, China). All animal procedures were approved by the Animal Ethics Committee of Fujian Medical University. Tumor‐bearing mice were prepared by subcutaneously injecting a suspension of the HepG2 cells (10^7^ cells) in sterilized 1 × PBS. When the tumor size reached about ≈100 mm^3^ and randomly divided into four groups, 200 µL of Ce6‐fDNA (Ce6, 5.12 × 10^−6^ m), Ce6‐fDNA^Dox^ (Ce6, 5.12 × 10^−6^ m; DOX, 98.87 × 10^−6^ m), and Ce6‐fNDNA^Dox^ (Ce6, 5.12 × 10^−6^ m; DOX, 98.87 × 10^−6^ m) were intravenous injected into HepG2‐bearing nude mice, respectively. One group of mice treated with the same volume of sterilized PBS was taken as the control. The mice were segregated into following four groups:

(1) Sterilized PBS with combined laser irradiation under 670 nm (0.2 W cm^−2^) for 10 min (*n* = 5);
(2) Ce6‐fDNA with laser irradiation under 670 nm (0.2 W cm^−2^) for 10 min (*n* = 5);
(3) Ce6‐fDNA^Dox^ with laser irradiation under 670 nm (0.2 W cm^−2^) for 10 min (*n* = 5);
(4) Ce6‐fNDNA^Dox^ with laser irradiation under 670 nm (0.2 W cm^−2^) for 10 min (*n* = 5).

For the in vivo fluorescence imaging experiments,^21^ the HepG2‐bearing nude mice were intravenously injected with Ce6‐fDNA^Dox^ or Ce6‐fNDNA^Dox^, and then were imaged with ChemiDoc MP Imaging System (Biorad). For the ex vivo fluorescence imaging experiments,^21^ HepG2‐bearing nude mice were intravenously injected with Ce6‐fDNA^Dox^ or Ce6‐fNDNA^Dox^, and then were sacrificed by cervical dislocation after 2 h of injection. After anatomization, the dissected organs, including tumor, heart, liver, spleen, lung, and kidney were imaged with ChemiDoc MP Imaging System (Biorad). For synergistic antitumor efficacy analysis, the laser irradiation was conducted after 2 h of intravenous injection. The therapeutic efficacy and toxicity were evaluated by monitoring the tumor volume changes with a Vernier caliper and body weight changes in each group every 2 d, up to 12 d. The tumor volume (*V*) was calculated using the following equation

(1) $$V=AB^{2}/2$$

where *A* and *B* are the longer and shorter diameter (mm) of the tumor, respectively. To further examine the histological changes of tumor, one tumor‐bearing mouse in each group was sacrificed after 2 d of irradiation, and the tumors were collected, and then stained with H&E for histopathology evaluation.

*Statistical Analysis*: Statistical analysis of data was performed using one‐way of variance (ANOVA) method or the two‐tailed paired Student's *t*‐test, *p < 0.05, **p < 0.01, ***p < 0.001, *p* < 0.05 was considered as statistically significant. All the data were shown as means ± SD through at least three experiments.

## Supporting information

As a service to our authors and readers, this journal provides supporting information supplied by the authors. Such materials are peer reviewed and may be re‐organized for online delivery, but are not copy‐edited or typeset. Technical support issues arising from supporting information (other than missing files) should be addressed to the authors.

Supplementary
