# Supplementary material for: Tumor Microenvironment Activable Self‐Assembled DNA Hybrids for pH and Redox Dual‐Responsive Chemotherapy/PDT Treatment of Hepatocellular Carcinoma
Source: Adv Sci (Weinh). 2017 Jan 24;4(4):1600460. doi: 10.1002/advs.201600460 (PMC5396159; doi:10.1002/advs.201600460)
Supplement: Supplementary file 1 — Supplementary [file ADVS-4-na-s001.pdf]

Copyright WILEY-VCH Verlag GmbH & Co. KGaA, 69469 Weinheim, Germany, 2017.

ADVANCED SCIENCE
Open Access

Supporting Information

for *Adv. Sci.*, DOI: 10.1002/advs.201600460

Tumor Microenvironment Activable Self-Assembled DNA Hybrids for pH and Redox Dual-Responsive Chemotherapy/PDT Treatment of Hepatocellular Carcinoma

Da Zhang, Aixian Zheng, Juan Li, Ming Wu, Zhixiong Cai, Lingjie Wu, Zuwu Wei, Huanghao Yang, Xiaolong Liu, and Jingfeng Liu**

Copyright WILEY-VCH Verlag GmbH & Co. KGaA, 69469 Weinheim, Germany, 2016.

Supporting Information

Tumor micro-environment activable self-assembled DNA hybrids for pH and redox dual-responsive chemotherapy / PDT treatment of Hepatocellular carcinoma

Da Zhang, Aixian Zheng, Juan Li, Ming Wu, Zhixiong Cai, Lingjie Wu, Zuwu Wei, Huanghao Yang, Xiaolong Liu, Jingfeng Liu*.*

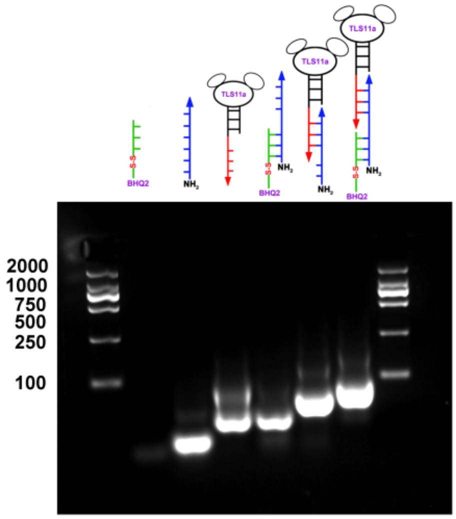

Figure S1. (A) Lane 1 and 8 is the DNA marker, lanes 2 to 4 are RQD, CD and TD, respectively. Lane 5, 6 is RQD + CD (without Ce6) and CD (without Ce6) + TD, respectively. Lane 7 was the self-assembled final products containing RQD, CD (without Ce6) and TD.

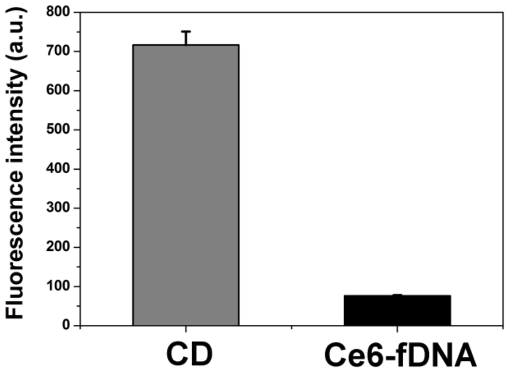

Figure S2. The mean fluorescence intensity (MFI) of Ce6 in CD or Ce6-fDNA as indicated (n = 3).

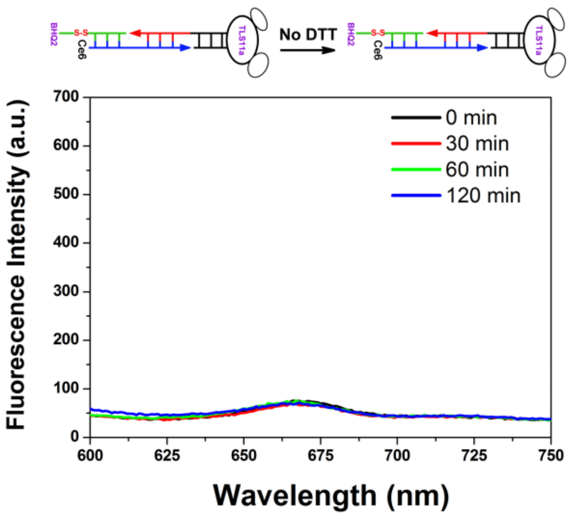

Figure S3. Fluorescence emission spectrum of Ce6-fDNA probe (excitation at 405 nm) in the absence of DTT (10 mM).

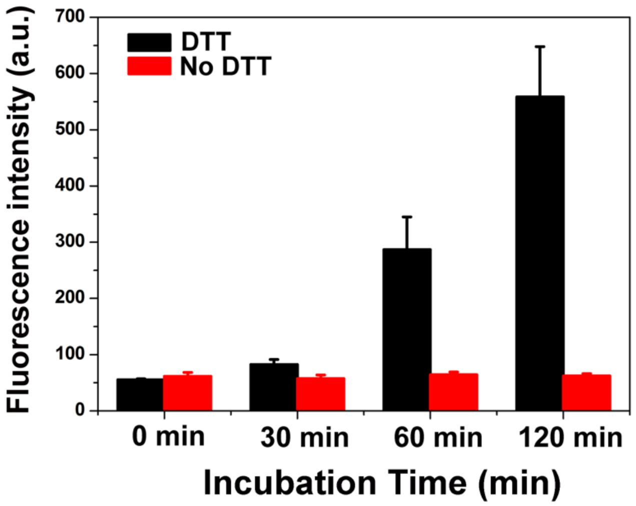

Figure S4. The mean fluorescence intensity (MFI) of Ce6 in Ce6-fDNA with (black) or without DTT (red) as indicated (excitation 405 nm, emission 670 nm) (n = 3).

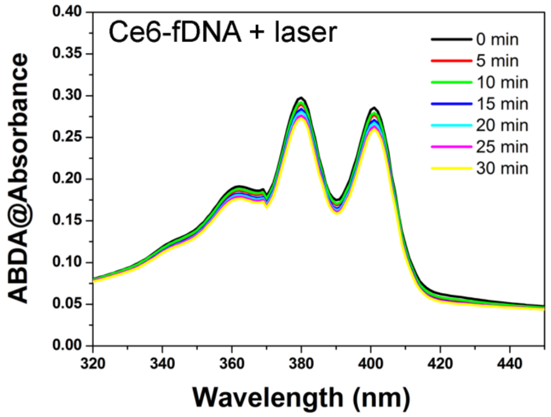

Figure S5. Absorbance of 9, 10-dimethylanthracene (ABDA, 100 μM) after photodecomposition by ROS generation upon NIR laser irradiation (670 nm (0.2 W/cm^2)) in the presence of the Ce6-fDNA only in TM buffer.

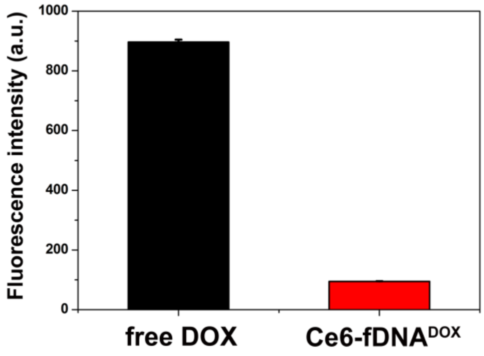

Figure S6. The mean fluorescence intensity (MFI) of Dox in free DOX and in Ce6-fDNA^{DOX} as indicated (excitation 488 nm, emission 595 nm) (n = 3).

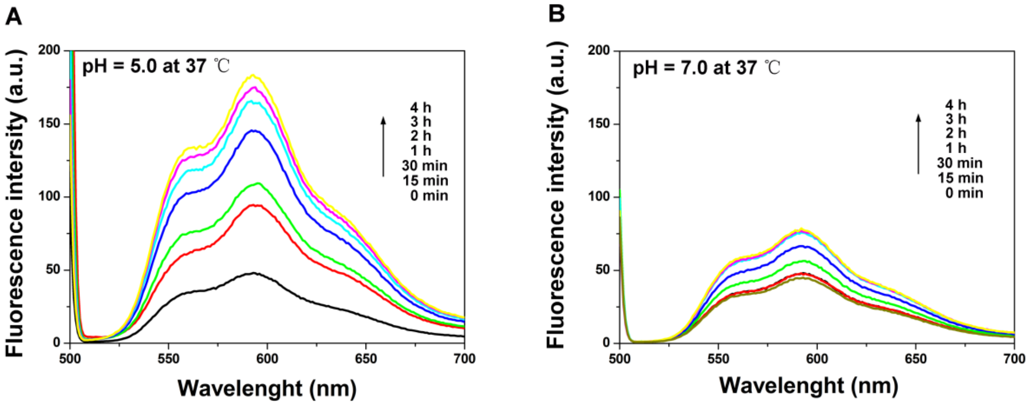

Figure S7. Fluorescence spectra of DOX were used to determine the release of doxorubicin from the Ce6-fDNA^{DOX} triggered by low pH value.

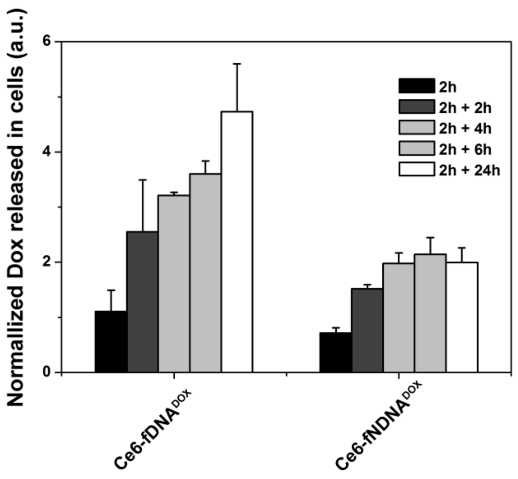

Figure S8. HepG2 cells incubated with Ce6-fDNA^{DOX} probe or Ce6-fNDNA^{DOX} (Ce6, 2.05 μM; Dox, 39.5 μM) for 2 h, 2h + 2h, 2h + 4h, 2h + 6h and 2h + 24h, respectively (n =5).
